# Efficacy and Safety of Transcranial Direct Current Stimulation for Attention Deficit Hyperactivity Disorder: A Meta–Analysis

**DOI:** 10.31083/AP47294

**Published:** 2025-10-20

**Authors:** Liqiong Wang, Wenjing Liao, Rongwang Yang

**Affiliations:** ^1^Mental Health Center, Children’s Hospital, Zhejiang University School of Medicine, National Clinical Research Center for Child Health, 310052 Hangzhou, Zhejiang, China

**Keywords:** transcranial direct current stimulation, attention deficit hyperactivity disorder, meta–analysis, adverse event

## Abstract

**Background:**

Attention deficit hyperactivity disorder (ADHD) is one of the most common neurodevelopmental disorders in children. Treatment strategies include psychotherapy, medication, education, and individual support. Recently, transcranial direct current stimulation (tDCS) has emerged as a potential therapeutic option. We undertook this meta–analysis and systematic review to evaluate the efficacy and safety of tDCS for ADHD.

**Methods:**

The PubMed, Embase, Cochrane Library, and Web of Science databases were systematically searched for randomized controlled trials (RCTs) assessing the efficacy of tDCS for ADHD. The search terms included “transcranial direct current stimulation” and “attention deficit hyperactivity disorder”. The search was conducted with no language restrictions, up to the deadline of December 1, 2024. Impulsivity symptoms, inattention, adverse events, and correct responses were analyzed using Stata 15.0.

**Results:**

Seven studies with 290 patients were included. The results of this meta–analysis indicated that tDCS reduced impulsive symptoms [standardized mean difference (SMD) = –0.60, 95% CI (–1.04, –0.16)] as well as inattentive symptoms [SMD = –1.00, 95% CI (–1.95, –0.04)] in patients with ADHD, and did not increase adverse effects [odds ratio (OR) = 1.26, 95% CI (0.67, 2.38)].

**Conclusions:**

tDCS can improve impulsive symptoms and inattentive symptoms in ADHD patients without increasing adverse effects, which is critical in clinical practice, especially when considering non–invasive brain stimulation. The study provided quantitative evidence that tDCS can be used for treating ADHD symptoms without adverse events.

**The PROSPERO Registration:**

This study was registered in PROSPERO (CRD42023451277), https://www.crd.york.ac.uk/PROSPERO/view/CRD42023451277.

## Main Points

1. Efficacy of transcranial direct current stimulation (tDCS) in reducing attention deficit hyperactivity disorder (ADHD) Symptoms: tDCS significantly reduced impulsive and inattentive symptoms in children with ADHD, with standardized mean differences (SMD) of –0.60 and –1.00, respectively.

2. Safety Profile of tDCS: tDCS did not lead to an increase in adverse events, with an odds ratio (OR) of 1.26, suggesting that it is a safe treatment option for ADHD.

3. The study highlights the potential of tDCS as a viable therapeutic option for ADHD, especially given its ability to improve symptoms without increasing adverse effects, making it an attractive choice in clinical practice.

## 1. Introduction

Attention deficit hyperactivity disorder (ADHD) is one of the most prevalent neurodevelopmental disorders in children [1]. The estimated prevalence of ADHD is 5.9% to 7.1% in children and youngsters according to the diagnostic criterion of the Diagnostic and Statistical Manual of Mental Disorders, Fourth Edition (DSM–IV) [2, 3]. The main ADHD symptoms include impulsivity, inattention, hyperactivity, cognitive deficits, executive functioning deficits, learning difficulties, and affective–emotional disorders [4, 5]. For children with normal or near–normal intelligence, ADHD has a serious impact on their academic performance, self–esteem, and self–confidence with peers, leading to a range of adverse health outcomes [6, 7]. ADHD has now become a global health problem and imposes a serious pressure on society and families [8, 9].

Treatment strategies of ADHD include medication, behavioral therapy, dietary therapy, and special education [10, 11]. Central stimulants are now the first–line drug for ADHD. Some patients may be not responsive to central stimulants and potential side effects have been reported such as appetite suppression and abdominal discomfort. These side effects might contribute to the discontinued administration of central stimulants. Cognitive behavioral therapy (CBT) remains one of the most effective psychotherapeutic treatments for people with ADHD; however, treatment with CBT alone has limited efficacy. Recent study has increasingly focused on non–pharmacological treatments, including transcranial direct current stimulation (tDCS). tDCS has shown the potential to enhance the efficacy of medications and improve treatment outcomes [12].

tDCS uses a weak electric current (1.0–2.0 mA) to modulate brain activity by applying electrodes on the scalp, with the current flowing from the anode to the target area and out through the cathode [13]. Anodal stimulation enhances neuronal activity and synaptic connections, while cathodic stimulation generally inhibits neuronal activity, though its effects may vary depending on the target brain region and its neural circuitry [14]. Meanwhile, the change of the excitableness by tDCS is affected by many factors, including current intensity, stimulation time, and electrode morphology [15, 16]. tDCS can only stimulate cortical areas and, as the dorsolateral prefrontal cortex (DLPFC) is an important part of the prefrontal cortex for cognitive network control, the DLPFC is often chosen as the target stimulation area for ADHD [17]. Jacobson* et al*. [18] found that tDCS stimulation of the inferior frontal gyrus (IFG) region can improve subjects’ response inhibition so this region is regarded as one of the target regions for ADHD therapy.

Currently, there is controversy regarding the efficiency of tDCS in the ADHD population [19]. Allenby* et al*.’s [20] study concluded that stimulation of the left DLPFC with tDCS can improve impulsivity symptoms in ADHD, but Westwood* et al*. [21] concluded that multiple sessions of anodic tDCS treatment did not improve ADHD symptoms or cognitive performance. The present study aimed to resolve the controversy through meta–analysis, whilst attempting to clarify if tDCS is an appropriate therapy choice for ADHD treatment.

## 2. Methods

This systemic review is registered in the online PROSPERO (https://www.crd.york.ac.uk/PROSPERO/view/CRD42023451277) international prospective register of systemic reviews [22] of the National Institute for Health Research (CRD42023451277). The PRISMA 2020 checklist is included in the **Supplementary Material-PRISMA_2020 checklist**.

### 2.1 Inclusion and Exclusion Criteria

The included population were all diagnosed with ADHD using the criteria of DSM–5 or International Classification of Diseases, 10th Edition (ICD–10) [23]. tDCS was applied to the experimental group and sham stimulation was used in the control group. The primary result metrics were impulsivity symptoms, inattention, and adverse events, and the secondary outcome metrics were correct responses. The main randomized controlled trial was included in this study.

Exclusion criteria: (1) participants who did not meet the diagnostic criteria for ADHD in the DSM–5, ICD–10, or other relevant guidelines; (2) participants with a history of serious neurologic or psychiatric illness (e.g., schizophrenia, bipolar disorder, major depression); (3) conference abstracts, meta–analyses, systematic reviews, animal experiments; (4) full text not available; and (5) case reports.

### 2.2 Literature Retrieval

Randomized controlled trials on tDCS for ADHD were retrieved from the PubMed (https://pubmed.ncbi.nlm.nih.gov/), Embase (https://www.embase.com/), Cochrane Library (https://www.cochranelibrary.com/) , and Web of Science databases (https://access.clarivate.com/), with a search deadline of December 1, 2024, using the MeSH term combined with a free word: Transcranial Direct Current Stimulation and Attention Deficit Disorder with Hyperactivity. The specific search results are shown in **Supplementary Material 1**.

### 2.3 Data Extraction

Two authors rigorously screened the literature based upon predetermined inclusion and exclusion criteria. In case of disagreement, resolution was achieved through discussion or the opinion of a third person was sought to reach a consensus. Information extracted from the included studies included the following key details: authors, sample size (experimental and control groups), year, age, gender, intervention, country, duration of treatment, and outcome.

### 2.4 Risk of Bias

Two investigators independently assessed the risk of bias using the Cochrane Collaboration’s tools [24], categorizing it as low, unclear, or high. In case of disagreement, a third investigator was consulted to reach a consensus. The assessment covered seven areas: generation of random sequences (selection bias), allocation concealment (selection bias), blinding of implementers and participants (performance bias), blinding of outcome assessors (detection bias), completeness of outcome data (attrition bias), selective reporting of outcomes (reporting bias), and other potential sources of bias. Each study was evaluated individually according to these criteria. If a study fully met all the criteria, it was classified as having a “low risk” of bias, indicating high quality and minimal overall bias. If a study met some but not all criteria, it was categorized as having an “unclear risk”, suggesting a moderate potential for bias. If a study did not meet any of the criteria, it was classified as having a “high risk”, indicating a significant risk of bias and low quality.

### 2.5 Data Analysis

The data were statistically analyzed using Stata 15.0 software (Stata Corp, College Station, TX, USA). Heterogeneity between included research was evaluated by means of I^2^ values or Q–statistics. I^2^ values of 0%, 25%, 50%, and 75% indicate no heterogeneity, low heterogeneity, moderate heterogeneity, and high heterogeneity, respectively. If the I^2^ value was equal to or greater than 50%, a sensitivity analysis was performed, with the intention of detecting the robustness of the results. On the condition that the heterogeneity was lower than 50%, analyses were conducted using a fixed–effects model. The standardized mean difference (SMD) and 95% confidence interval (CI) were used for continuous variables; the odds ratio (OR) and 95% CI were used for dichotomous variables. In addition, the random effects model (REM) and Egger’s test were used for the purpose of evaluating publication bias.

## 3. Results

### 3.1 Study Selection

**Fig. 1.**
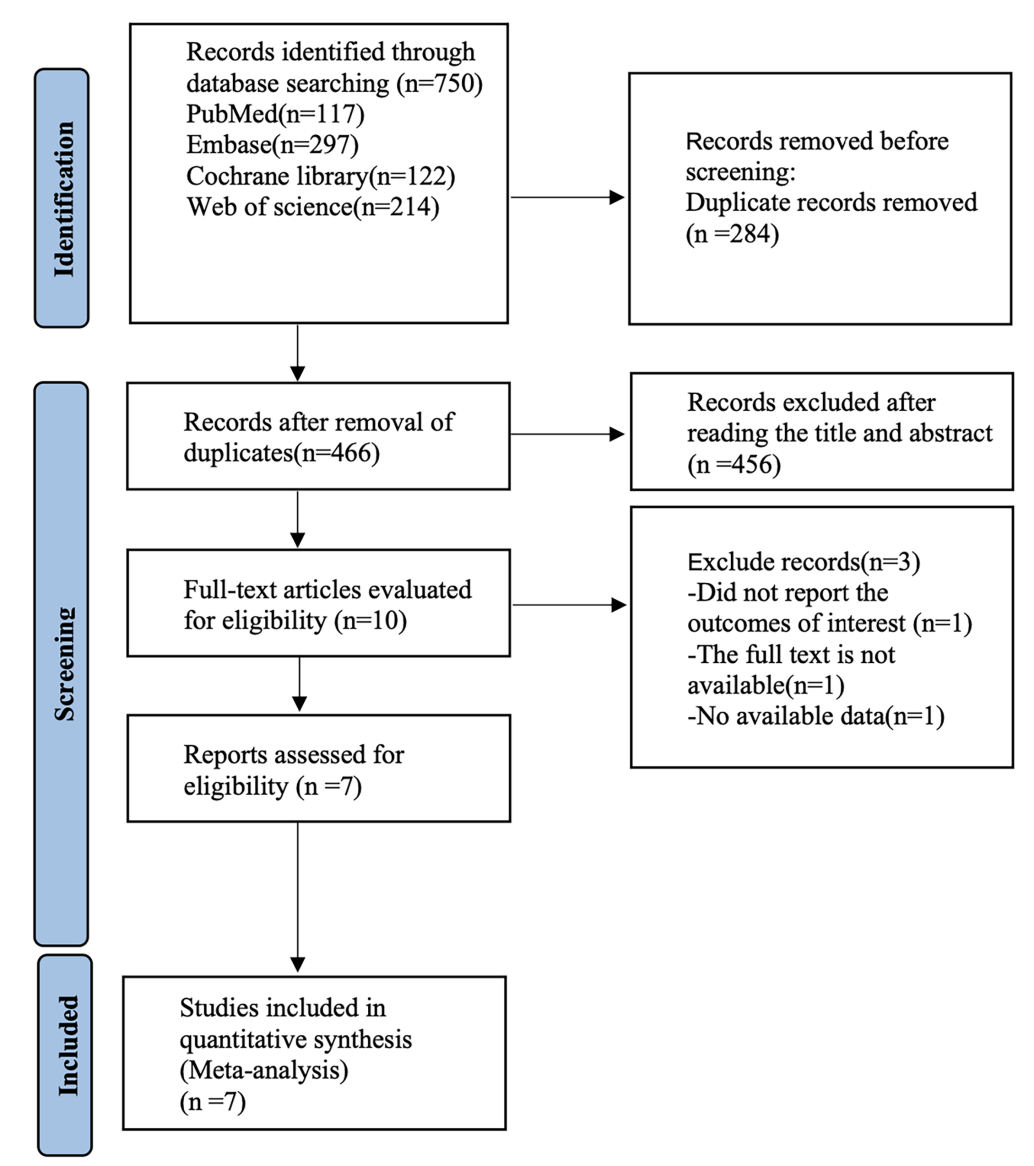
**Literature search process (Database established-December 1, 2024)**.

**Fig. 2.**
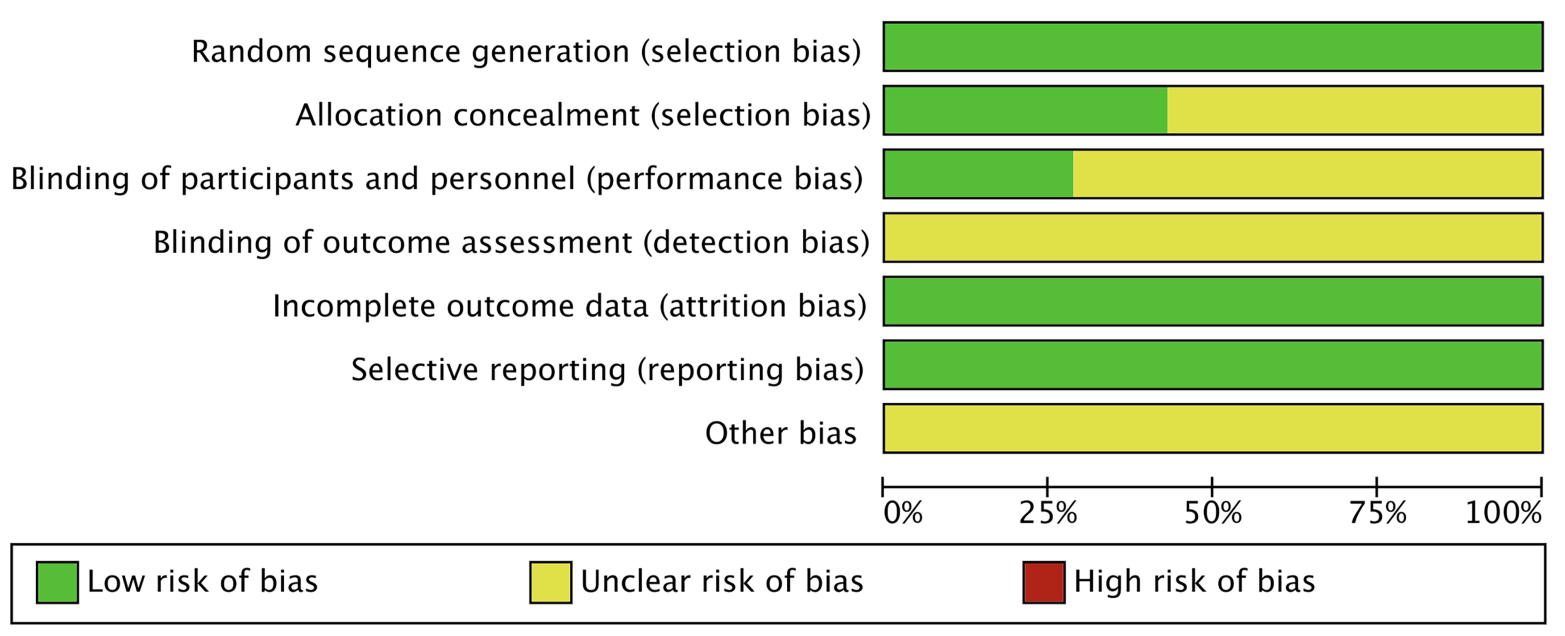
**Risk of bias**.

**Fig. 3.**
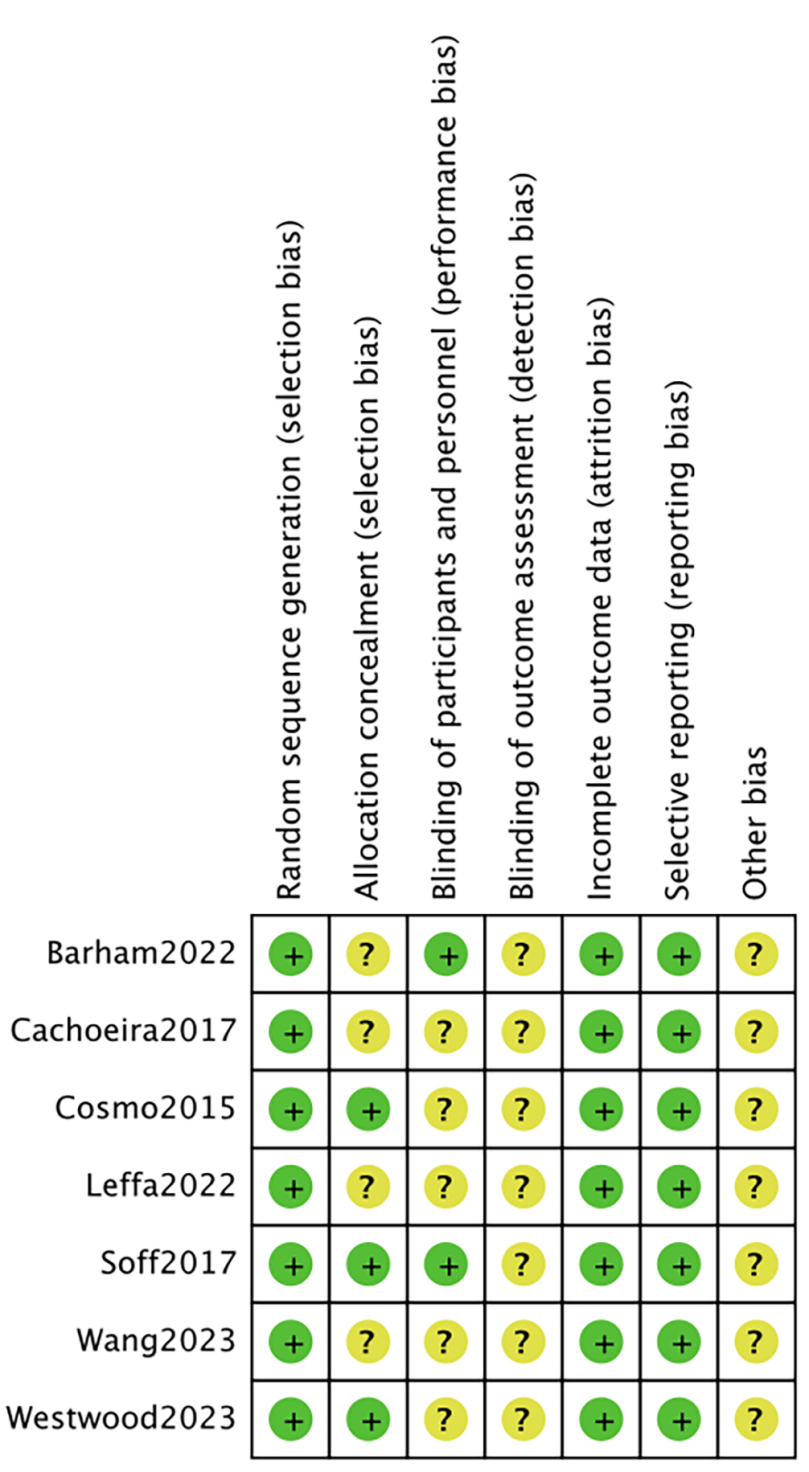
**Risk of bias summary. +: low risk; ?: high risk**.

The literature search procedure initially retrieved 750 articles; 284 duplicates were removed, 456 articles were removed after reading the titles and abstracts, 3 articles were removed after reading the full text, and finally 7 randomized controlled trials were included in our analysis (Fig. 1) [21, 25, 26, 27, 28, 29, 30]. Seven studies containing 290 patients were included, in which tDCS was mostly used with 1–2 mA stimulation at the DLPFC. Literature characteristics are shown in Table 1, Ref. [21, 25, 26, 27, 28, 29, 30]. Barham * et al*. [28], Cachoeira * et al*. [29], Cosmo * et al*. [27], Leffa *et al*. [30], Soff * et al*. [25], Wang * et al*. [26], and Westwood * et al*. [21] clearly explained the randomized method used, which was evaluated as low risk. Leffa * et al*. [30] and Barham * et al*. [28] were rated as low risk by double–blind evaluation. The risk of bias is shown in Fig. 2; Fig. 3, Ref. [21, 25, 26, 27, 28, 29, 30].

**Table 1.** **Literature characteristics**.

Study	Year	Country	Sample size		Mean age		Gender (M/F)	Intervention		Treatment time	Outcome
			EG	CG	EG	CG		EG	CG		
Barham * et al*. [28]	2022	Turkey	11	11	22.45	21.72	7/15	tDCS, 2 mA, 20 min, DLPFC	sham stimulation	NA	F1; F3
Cosmo * et al*. [27]	2015	Brazil	30	30	31.83	32.67	35/25	tDCS, 1 mA, 20 min, DLPFC	sham stimulation	NA	F1; F2; F3
Cachoeira * et al*. [29]	2017	Brazil	9	8	31	33.75	8/9	tDCS, 2 mA, 20 min, DLPFC	sham stimulation	5 days	F2; F3; F4; F5
Leffa * et al*. [30]	2022	Brazil	32	32	38.2	38.4	34/30	tDCS, 2 mA, 30 min, DLPFC	sham stimulation	28 days	F2; F5
Soff * et al*. [25]	2017	Germany	15	15	14.2	14.2	6/24	tDCS, 2 mA, 20 min, DLPFC	sham stimulation	5 days	F2; F3; F5
Wang * et al*. [26]	2023	China	24	23	11.29	11.37	27/20	tDCS, 1 mA, 20 min, DLPFC	sham stimulation	5 days	F1
Westwood * et al*. [21]	2023	UK	24	26	13.05	14.23	NA	tDCS, 1 mA, 20 min, DLPFC	sham stimulation	15 days	F2; F3; F5

EG, experimental group; CG, control group; DLPFC, dorsolateral prefrontal cortex; NA, not reported; F1, correct responses; F2, impulsivity symptoms; F3, inattention; F4, sheehan disability scale; F5, adverse events; tDCS, transcranial direct current stimulation.

### 3.2 Results of Meta–Analysis

#### 3.2.1 Impulsivity Symptoms

**Fig. 4.**
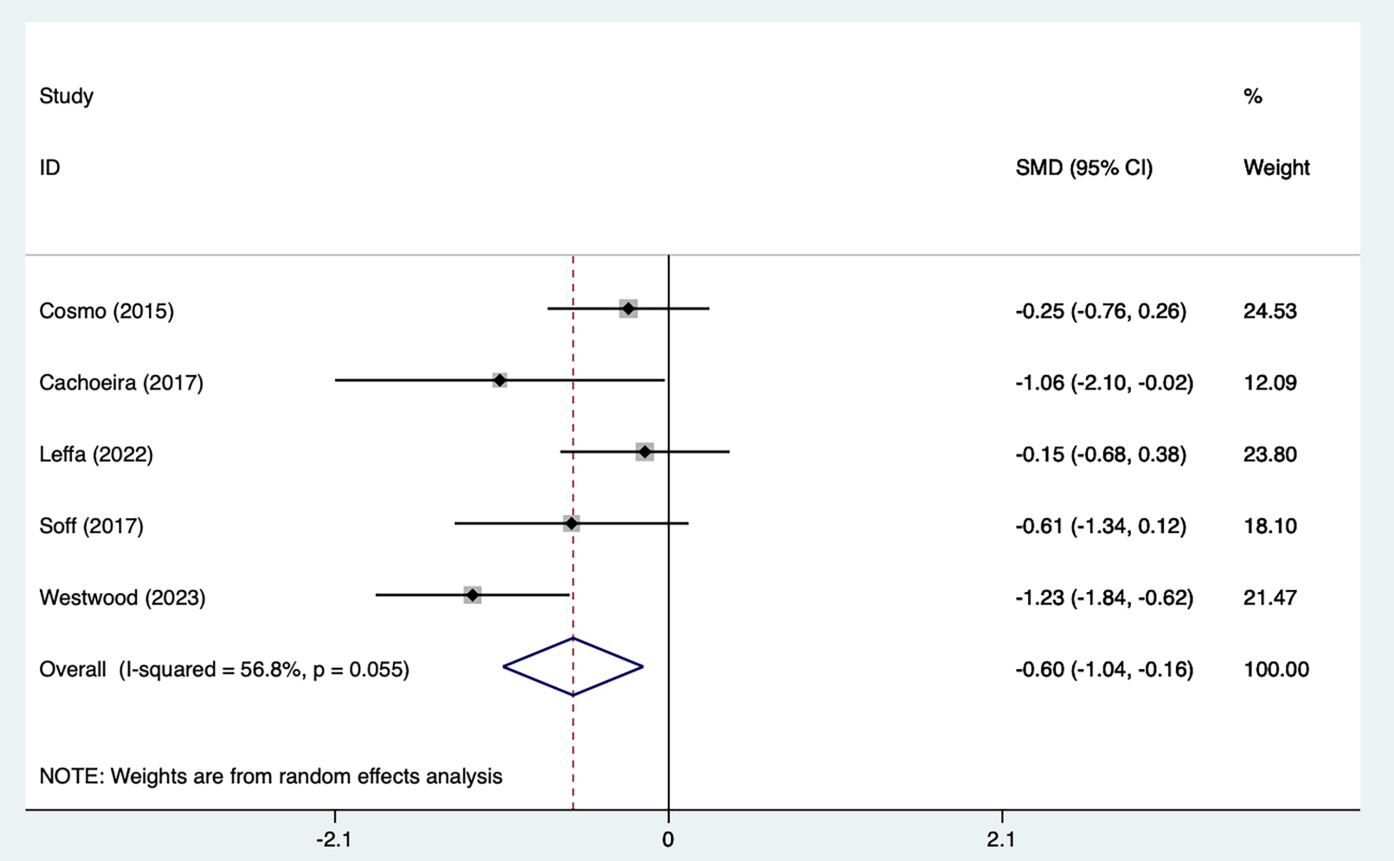
**Forest plot of meta–analysis of impulsive symptoms**. SMD, standardized mean difference; CI: confidence interval.

**Fig. 5.**
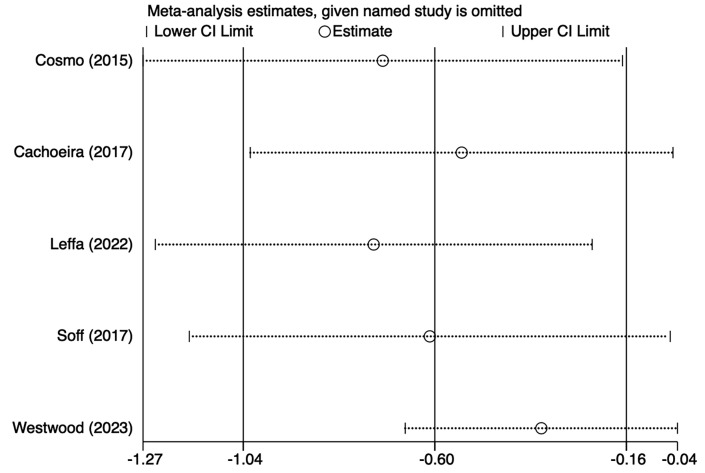
**Sensitivity analysis of impulsive symptoms**.

Five studies [21, 25, 27, 29, 30] mentioned impulsivity symptoms, including 103 in the tDCS groups and 104 in the sham stimulation groups. Fig. 4, Ref. [21, 25, 27, 29, 30] shows the results of the heterogeneity test (I^2^ = 56.8%, *p* = 0.055). The analysis was implemented through the REM and the results [SMD = –0.60, 95% CI (–1.04, –0.16)] suggested that tDCS could reduce impulsivity symptoms in ADHD patients. Because of the heterogeneity, we used one–by–one exclusion for sensitivity analysis, suggesting that the analysis was more stable (Fig. 5, Ref. [21, 25, 27, 29, 30]).

#### 3.2.2 Inattention Symptoms

**Fig. 6.**
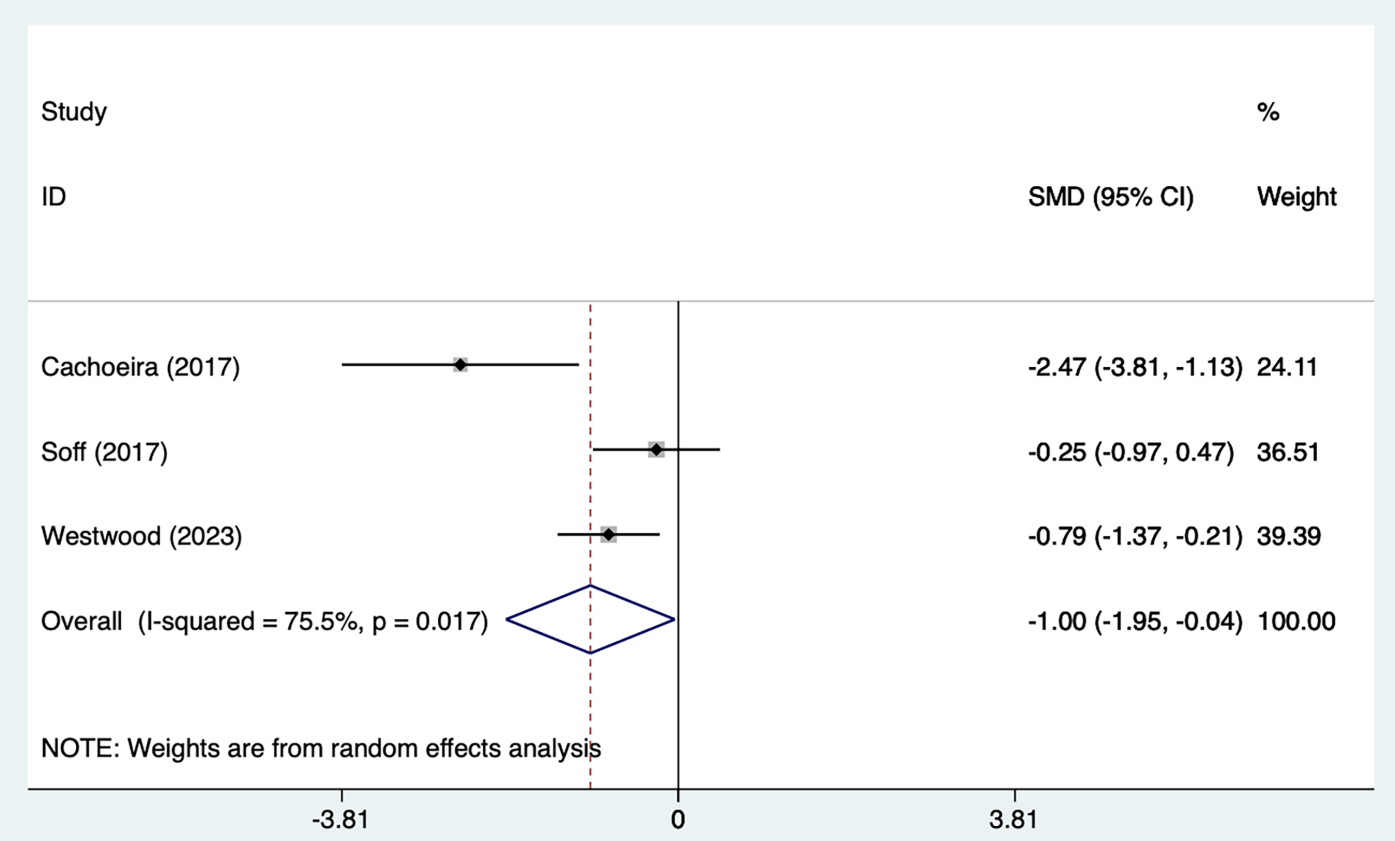
**Forest plot of meta–analysis of inattention symptoms**.

Fig. 6, Ref. [21, 25, 29] shows a forest plot summarizing the effect sizes and overall analysis results of the three studies. The results of the REM [SMD = –1.00, 95% CI (–1.95, –0.04)], indicating that the overall effect was significant. The heterogeneity was high (I^2^ = 75.5%, *p* = 0.017), suggesting that there was a large variation among studies, which may be affected by the heterogeneity of methods or samples. The key interpretation was that although the overall effect was significant, the lower limit of the confidence interval was close to 0 (–0.04) and the actual clinical significance should be evaluated with caution. The weight distribution showed that Westwood * et al*. [21] contributed most to the results.

#### 3.2.3 Correct Response Symptoms

**Fig. 7.**
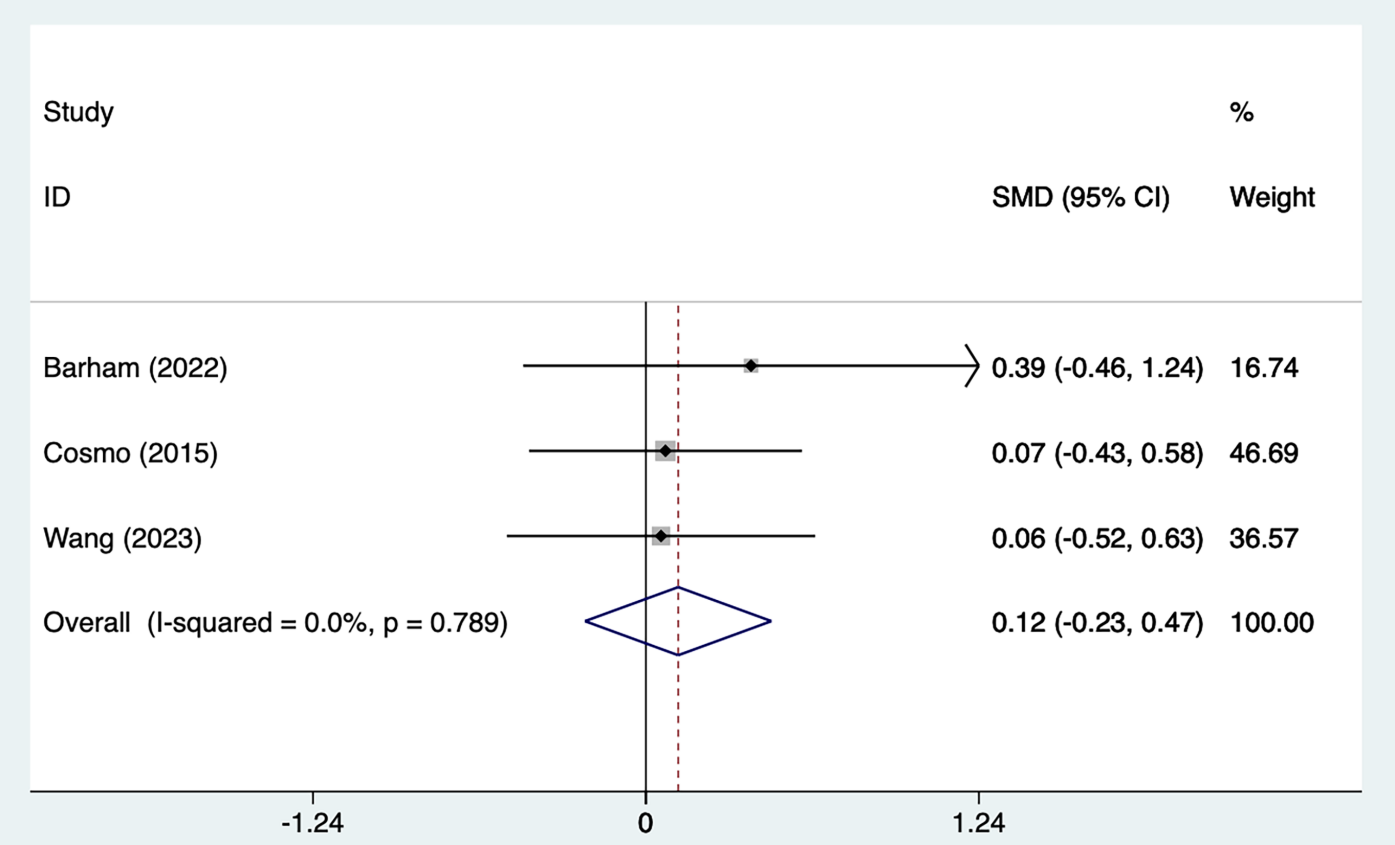
**Forest plot of meta–analysis of correct response symptoms**.

Three studies [26, 27, 28] mentioned correct response symptoms, including 65 in the tDCS groups and 64 in the sham stimulation groups. Fig. 7, Ref. [26, 27, 28] shows the results of the test of heterogeneity (I^2^ = 0%, *p* = 0.789). The analysis was implemented through the fixed effects model (FEM). The results [SMD = 0.12, 95% CI (–0.23, 0.47)] suggested that tDCS did not significantly improve correct response symptoms in ADHD patients.

#### 3.2.4 Adverse Events

**Fig. 8.**
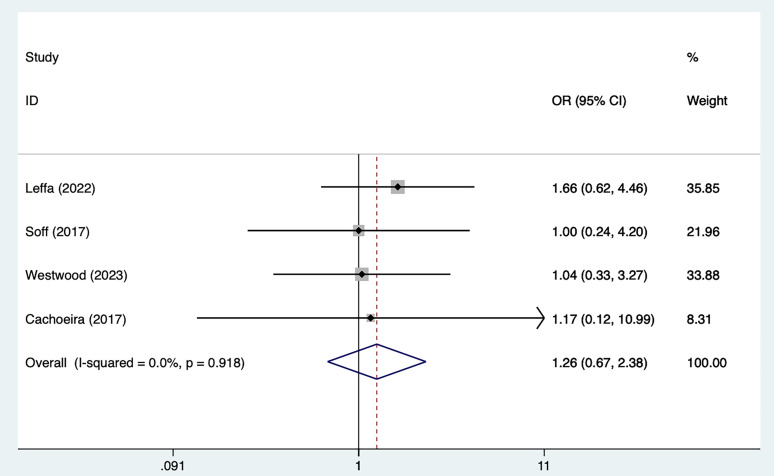
**Forest plot of meta–analysis of adverse events**. OR, odds ratio.

Four studies [21, 25, 29, 30] mentioned adverse events, including 79 in the tDCS groups and 82 in the sham stimulation groups. Fig. 8, Ref. [21, 25, 29, 30] shows the results of no heterogeneity according to the test of heterogeneity (I^2^ = 0%, *p* = 0.918). The analysis was executed through the FEM and the results [OR = 1.26, 95% CI (0.67, 2.38)] suggested that tDCS did not significantly improve adverse events in ADHD patients.

#### 3.2.5 Published Bias

**Fig. 9.**
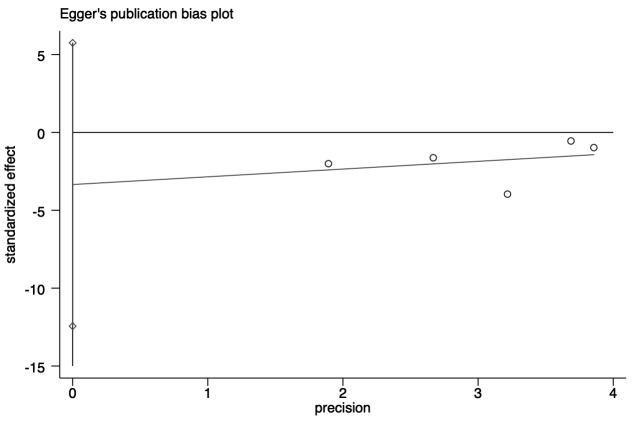
**Egger test of impulsive symptoms**.

Publication bias was evaluated using Egger’s test (*p* = 0.523) for impulsive symptoms. Fig. 9 shows the results of the Egger’s test (the X–axis represents the precision, the Y–axis standardizes effect size, and the different study are located on both sides of the line, representing the absence of heterogeneity).

## 4. Discussion

### 4.1 Effects of Stimulation Mode on ADHD Symptoms

Our study shows that after tDCS intervention, impulsivity symptoms and inattention symptoms of ADHD patients are substantially improved with no increase in adverse effects. Our discoveries correspond to those of Sotnikova* et al*. [31], who assessed the efficacy of tDCS for ADHD using the German Fragebogen zur Beurteilung von Verhaltensstörungen bei Kindern und Jugendlichen (FBB)–ADHD–Skala Diagnostic Scale, which showed an improvement in clinical symptoms and the efficacy of 5–day tDCS treatment that lasted for at least 10 days. Research by Nejati* et al*. [32]. further explored the effects of different stimulation modes on executive function and showed that both anodal and cathodal stimulation in the left DLPFC improved subjects’ executive function, with anodal stimulation showing significantly better results than cathodal stimulation. Specifically, cathodal stimulation mainly improved response inhibition, whereas anodal stimulation was able to improve working memory, reaction time, and task accuracy. Furthermore, the combination of anodal stimulation of the left DLPFC with cathodal stimulation of the right DLPFC, although having no significant effect on response inhibition, showed better results in improving task accuracy and improving working memory. The results of our study further support this finding. In particular, the choice of anode/cathode stimulation plays an important role in the improvement of ADHD symptoms under different electrode configurations. Compared with cathodal stimulation, anodal stimulation showed more significant effects on improving executive function and response inhibition. In addition, differences in electrode placement and stimulation intensity may also affect the efficacy of tDCS, which varied significantly across studies.

### 4.2 Effects of Stimulus Mode and Task Type

In addition, the task setting during stimulation seems to have an important influence on the effects of tDCS. Task interventions, such as response inhibition tasks and working memory tasks, were set up during stimulation, and the impact of these tasks on the generation of stimulation effects may not be negligible. For example, in our study, ADHD patients who received F8 anodic stimulation showed improved performance on a working memory task, with shorter reaction times and improved task accuracy. However, the effect of combined anodic and cathodic stimulation becomes more complex when the task setting focuses more on response inhibition. Different tasks require different executive functions, which may lead to different patterns of effects.

Another clinical controlled study [33] investigated 42 ADHD patients aged 13–17 years old. The left DLPFC anodic stimulus showed a significant effect when combined with a response inhibition task, whereas the combination of anodic and cathodic stimuli in a working memory task significantly shortened reaction times and improved task accuracy, despite no significant improvement in response inhibition. In our study, we found that differences in task type alter the effect of the stimuli. In response inhibition tasks, anodic stimulation improved symptoms more significantly in ADHD patients, whereas in working memory tasks, anodic stimulation combined with cathodic stimulation may have been more effective in improving task accuracy and working memory capacity. These results suggest that the selection of stimulation modes not only needs to consider the specific symptoms of ADHD but should also be optimized in conjunction with the type of task. We suggest that in the clinical application of tDCS for ADHD, task interventions should be carefully designed and the cooperation of different stimulation modes should be considered to obtain the best therapeutic effect.

### 4.3 Inconsistency of Results 

Although most existing studies [34, 35] generally agree that tDCS can significantly improve ADHD symptoms, especially in terms of improving the rate of correct responses, our study failed to observe an enhancement of this effect. We believe that this inconsistency may be related to several factors. First, the small sample size may have led to insufficient statistical power. Second, there are differences in stimulation protocols between studies, including current intensity, electrode placement, electrode size, and stimulation mode, which may have an impact on the effects of tDCS. In addition, differences in task settings may also be an important factor contributing to inconsistent results. For example, some studies used a simpler response inhibition task, while others used a more complex working memory task, which may have led to differences in the effects of tDCS across tasks [36, 37].

Therefore, future studies should use larger sample sizes, standardize stimulation protocols, and further explore the interaction between task type and stimulation mode to more accurately evaluate the efficacy of tDCS in the treatment of ADHD.

### 4.4 Safety Analysis

No serious side effects requiring medical intervention were reported for using tDCS in ADHD. Side effects were mostly mild and self–limited, including primarily local itching, tingling, erythema, and local burning, as well as headache and neck pain. This suggests that tDCS treatment is safe and can be well tolerated by children with ADHD.

### 4.5 Strengths and Limitations

Compared with a previously published meta–analysis [38], our study has the following advantages. Firstly, the current study used more stringent screening criteria and included only randomized controlled trials, while the previous meta–analysis included crossover trials, which would lead to a decrease in the credibility of the study findings. Secondly, our study collected the latest randomized controlled trials. After tDCS intervention, impulsivity symptoms and inattention symptoms of ADHD patients were substantially improved without increased adverse effects. Compared with Salehinejad * et al*. [39], this study focused solely on ADHD and conducted a quantitative analysis of the outcomes.

There still exist some constraints in our study. Firstly, the small number of included studies and the small sample sizes involved lead us to be cautious about the conclusions of the study. Secondly, the intensity of the tDCS used was not the same in each study and the duration of the treatment was not the same, which is a possible source of heterogeneity. Thirdly, environmental factors (e.g., background noise, lighting, and participant engagement during stimulation) may have varied from setting to setting, and these factors may have influenced the outcome of tDCS interventions.

In addition, the type and difficulty of cognitive tasks performed during stimulation (e.g., attention or memory tasks) varied across studies, which may have contributed to the differences in observed efficacy. In addition, individual differences, including age, ADHD subtype (e.g., inattentive vs mixed), and baseline symptom severity, may also influence response to tDCS. These factors were not consistently controlled for in the included studies; therefore, we should be cautious in interpreting these results.

## 5. Conclusions

Typical symptoms of ADHD, such as impulsivity and attention deficit, were highlighted in this study. The study provides quantitative evidence regarding the treatment of ADHD symptoms with tDCS. tDCS may improve impulsive symptoms and inattentive symptoms among ADHD patients without increasing adverse effects, which is critical for clinical practice, especially when considering non–invasive brain stimulation, where patient safety is a key concern.

## Data Availability

All the data used in this manuscript have been included in the tables and figures.

## Abbreviations

tDCS, transcranial direct current stimulation; ADHD, attention deficit hyperactivity disorder; DLPFC, dorsolateral prefrontal cortex; SMD, standardized mean difference; CI, confidence interval; OR, odds ratio.

## Supplementary Material

Supplementary material associated with this article can be found, in the online version, at https://doi.org/10.31083/AP47294.

## References

[1] Rajaprakash M, Leppert ML (2022). Attention–Deficit/Hyperactivity Disorder. *Pediatrics in Review*.

[2] Willcutt EG (2012). The prevalence of DSM–IV attention–deficit/hyperactivity disorder: a meta–analytic review. *Neurotherapeutics*.

[3] Pan G, Han Y, Wang TC, Chen ZY, Wang XQ, Sun HB (2024). Attention deficit hyperactivity disorder in children with epilepsy: a multicenter cross–sectional analysis in China. *World Journal of Pediatrics*.

[4] Dalsgaard S (2021). More Evidence Linking Autoimmune Diseases to Attention–Deficit/Hyperactivity Disorder. *JAMA Pediatrics*.

[5] Loe IM, Kakar PA, Sanders LM (2021). Diagnosis, Evaluation, and Treatment of Attention–Deficit/Hyperactivity Disorder. *JAMA Pediatrics*.

[6] Bell ZE, Fristad MA, Youngstrom EA, Arnold LE, Beauchaine TP, LAMS Consortium (2022). Attention–Deficit/Hyperactivity Disorder Symptoms and Externalizing Progression in the LAMS Study: A Test of Trait Impulsivity Theory. *Journal of the American Academy of Child and Adolescent Psychiatry*.

[7] Suarez EA, Bushnell GA (2022). Association Between Attention–Deficit/Hyperactivity Disorder and Benzodiazepines and Z–Hypnotics in Pregnancy–Questions Remain. *JAMA Network Open*.

[8] Shen L, Wang C, Tian Y, Chen J, Wang Y, Yu G (2021). Effects of Parent–Teacher Training on Academic Performance and Parental Anxiety in School–Aged Children With Attention–Deficit/Hyperactivity Disorder: A Cluster Randomized Controlled Trial in Shanghai, China. *Frontiers in Psychology*.

[9] Kim S, Hwang J, Lee JH, Park J, Kim HJ, Son Y (2024). Psychosocial alterations during the COVID–19 pandemic and the global burden of anxiety and major depressive disorders in adolescents, 1990–2021: challenges in mental health amid socioeconomic disparities. *World Journal of Pediatrics*.

[10] Michelini G, Norman LJ, Shaw P, Loo SK (2022). Treatment biomarkers for ADHD: Taking stock and moving forward. *Translational Psychiatry*.

[11] Montaleão Brum Alves R, Ferreira da Silva M, Assis Schmitz É, Juarez Alencar A (2022). Trends, Limits, and Challenges of Computer Technologies in Attention Deficit Hyperactivity Disorder Diagnosis and Treatment. *Cyberpsychology, Behavior and Social Networking*.

[12] Cosmo C, DiBiasi M, Lima V, Grecco LC, Muszkat M, Philip NS (2020). A systematic review of transcranial direct current stimulation effects in attention–deficit/hyperactivity disorder. *Journal of Affective Disorders*.

[13] Rubia K, Westwood S, Aggensteiner PM, Brandeis D (2021). Neurotherapeutics for Attention Deficit/Hyperactivity Disorder (ADHD): A Review. *Cells*.

[14] Nitsche MA, Paulus W (2000). Excitability changes induced in the human motor cortex by weak transcranial direct current stimulation. *The Journal of Physiology*.

[15] Lipka R, Ahlers E, Reed TL, Karstens MI, Nguyen V, Bajbouj M (2021). Resolving heterogeneity in transcranial electrical stimulation efficacy for attention deficit hyperactivity disorder. *Experimental Neurology*.

[16] Sousa B, Martins J, Castelo–Branco M, Gonçalves J (2022). Transcranial Direct Current Stimulation as an Approach to Mitigate Neurodevelopmental Disorders Affecting Excitation/Inhibition Balance: Focus on Autism Spectrum Disorder, Schizophrenia, and Attention Deficit/Hyperactivity Disorder. *Journal of Clinical Medicine*.

[17] Yang D, Shin YI, Hong KS (2021). Systemic Review on Transcranial Electrical Stimulation Parameters and EEG/fNIRS Features for Brain Diseases. *Frontiers in Neuroscience*.

[18] Jacobson L, Ezra A, Berger U, Lavidor M (2012). Modulating oscillatory brain activity correlates of behavioral inhibition using transcranial direct current stimulation. *Clinical Neurophysiology*.

[19] Pagán AF, Huizar YP, Short TR, Gotcher Z, Schmidt AT (2023). Adult Attention–Deficit/Hyperactivity Disorder: a Narrative Review of Biological Mechanisms, Treatments, and Outcomes. *Current Neurology and Neuroscience Reports*.

[20] Allenby C, Falcone M, Bernardo L, Wileyto EP, Rostain A, Ramsay JR (2018). Transcranial direct current brain stimulation decreases impulsivity in ADHD. *Brain Stimulation*.

[21] Westwood SJ, Criaud M, Lam SL, Lukito S, Wallace–Hanlon S, Kowalczyk OS (2023). Transcranial direct current stimulation (tDCS) combined with cognitive training in adolescent boys with ADHD: a double–blind, randomised, sham–controlled trial. *Psychological Medicine*.

[22] Page MJ, McKenzie JE, Bossuyt PM, Boutron I, Hoffmann TC, Mulrow CD (2021). The PRISMA 2020 statement: an updated guideline for reporting systematic reviews. *BMJ (Clinical Research Ed.)*.

[23] Koyuncu A, Ayan T, Ince Guliyev E, Erbilgin S, Deveci E (2022). ADHD and Anxiety Disorder Comorbidity in Children and Adults: Diagnostic and Therapeutic Challenges. *Current Psychiatry Reports*.

[24] Higgins JPT, Altman DG, Gøtzsche PC, Jüni P, Moher D, Oxman AD (2011). The Cochrane Collaboration’s tool for assessing risk of bias in randomised trials. *BMJ (Clinical Research Ed.)*.

[25] Soff C, Sotnikova A, Christiansen H, Becker K, Siniatchkin M (2017). Transcranial direct current stimulation improves clinical symptoms in adolescents with attention deficit hyperactivity disorder. *Journal of Neural Transmission*.

[26] Wang YC, Liu J, Wu YC, Wei Y, Xie HJ, Zhang T (2023). A randomized, sham–controlled trial of high–definition transcranial direct current stimulation on the right orbital frontal cortex in children and adolescents with attention–deficit hyperactivity disorder. *Frontiers in Psychiatry*.

[27] Cosmo C, Baptista AF, de Araújo AN, do Rosário RS, Miranda JGV, Montoya P (2015). A Randomized, Double–Blind, Sham–Controlled Trial of Transcranial Direct Current Stimulation in Attention–Deficit/Hyperactivity Disorder. *PLoS ONE*.

[28] Barham H, Büyükgök D, Aksu S, Soyata AZ, Bulut G, Eskicioğlu G (2022). Evidence for modulation of planning and working memory capacities by transcranial direct current stimulation in a sample of adults with attention deficit hyperactivity disorder. *Neuroscience Letters*.

[29] Cachoeira CT, Leffa DT, Mittelstadt SD, Mendes LST, Brunoni AR, Pinto JV (2017). Positive effects of transcranial direct current stimulation in adult patients with attention–deficit/hyperactivity disorder – A pilot randomized controlled study. *Psychiatry Research*.

[30] Leffa DT, Grevet EH, Bau CHD, Schneider M, Ferrazza CP, da Silva RF (2022). Transcranial Direct Current Stimulation vs Sham for the Treatment of Inattention in Adults With Attention–Deficit/Hyperactivity Disorder: The TUNED Randomized Clinical Trial. *JAMA Psychiatry*.

[31] Sotnikova A, Soff C, Tagliazucchi E, Becker K, Siniatchkin M (2017). Transcranial Direct Current Stimulation Modulates Neuronal Networks in Attention Deficit Hyperactivity Disorder. *Brain Topography*.

[32] Nejati V, Salehinejad MA, Nitsche MA, Najian A, Javadi AH (2020). Transcranial Direct Current Stimulation Improves Executive Dysfunctions in ADHD: Implications for Inhibitory Control, Interference Control, Working Memory, and Cognitive Flexibility. *Journal of Attention Disorders*.

[33] Breitling C, Zaehle T, Dannhauer M, Bonath B, Tegelbeckers J, Flechtner HH (2016). Improving Interference Control in ADHD Patients with Transcranial Direct Current Stimulation (tDCS). *Frontiers in Cellular Neuroscience*.

[34] Sierawska A, Prehn–Kristensen A, Moliadze V, Krauel K, Nowak R, Freitag CM (2019). Unmet Needs in Children With Attention Deficit Hyperactivity Disorder–Can Transcranial Direct Current Stimulation Fill the Gap? Promises and Ethical Challenges. *Frontiers in Psychiatry*.

[35] Zhang K, Yuan J, Pei X, Fu Z, Zhao Y, Hu N (2023). Cerebral blood flow characteristics of drug–naïve attention–deficit/hyperactivity disorder with social impairment: Evidence for region–symptom specificity. *Frontiers in Neuroscience*.

[36] Koyun AH, Wendiggensen P, Roessner V, Beste C, Stock AK (2024). Effects of Catecholaminergic and Transcranial Direct Current Stimulation on Response Inhibition. *The international journal of neuropsychopharmacology*.

[37] Barham H, Büyükgök D, Aksu S, Soyata AZ, Bulut G, Eskicioğlu G (2022). Evidence for modulation of planning and working memory capacities by transcranial direct current stimulation in a sample of adults with attention deficit hyperactivity disorder. *Neuroscience letters*.

[38] Brauer H, Breitling–Ziegler C, Moliadze V, Galling B, Prehn–Kristensen A (2021). Transcranial direct current stimulation in attention–deficit/hyperactivity disorder: A meta–analysis of clinical efficacy outcomes. *Progress in Brain Research*.

[39] Salehinejad MA, Ghanavati E, Glinski B, Hallajian AH, Azarkolah A (2022). A systematic review of randomized controlled trials on efficacy and safety of transcranial direct current stimulation in major neurodevelopmental disorders: ADHD, autism, and dyslexia. *Brain and Behavior*.

