# Supplementary material for: Efficacy and Safety of Transcranial Direct Current Stimulation for Attention Deficit Hyperactivity Disorder: A Meta–Analysis
Source: Alpha Psychiatry. 2025 Oct 20;26(5):47294. doi: 10.31083/AP47294 (PMC12593796; doi:10.31083/AP47294)
Supplement: Supplementary file 1 [file 2757-8038-26-5-47294-s1.zip › Supplementary Material 1.docx]

PubMed

(("Transcranial Direct Current Stimulation"[Mesh]) OR (((((((((((((((((((((((((Transcranial Direct Current Stimulation[Title/Abstract]) OR (tDCS[Title/Abstract])) OR (Cathodal Stimulation Transcranial Direct Current Stimulation[Title/Abstract])) OR (Cathodal Stimulation tDCS[Title/Abstract])) OR (Cathodal Stimulation tDCSs[Title/Abstract])) OR (Stimulation tDCS, Cathodal[Title/Abstract])) OR (Stimulation tDCSs, Cathodal[Title/Abstract])) OR (tDCS, Cathodal Stimulation[Title/Abstract])) OR (tDCSs, Cathodal Stimulation[Title/Abstract])) OR (Transcranial Random Noise Stimulation[Title/Abstract])) OR (Transcranial Alternating Current Stimulation[Title/Abstract])) OR (Transcranial Electrical Stimulation[Title/Abstract])) OR (Electrical Stimulation, Transcranial[Title/Abstract])) OR (Electrical Stimulations, Transcranial[Title/Abstract])) OR (Stimulation, Transcranial Electrical[Title/Abstract])) OR (Stimulations, Transcranial Electrical[Title/Abstract])) OR (Transcranial Electrical Stimulations[Title/Abstract])) OR (Anodal Stimulation Transcranial Direct Current Stimulation[Title/Abstract])) OR (Anodal Stimulation tDCS[Title/Abstract])) OR (Anodal Stimulation tDCSs[Title/Abstract])) OR (Stimulation tDCS, Anodal[Title/Abstract])) OR (Stimulation tDCSs, Anodal[Title/Abstract])) OR (tDCS, Anodal Stimulation[Title/Abstract])) OR (tDCSs, Anodal Stimulation[Title/Abstract])) OR (Repetitive Transcranial Electrical Stimulation[Title/Abstract]))) AND (("Attention Deficit Disorder with Hyperactivity"[Mesh]) OR (((((((((((((((((((((((Attention Deficit Disorder with Hyperactivity[Title/Abstract]) OR (Attention Deficit Disorders with Hyperactivity[Title/Abstract])) OR (ADHD[Title/Abstract])) OR (Attention Deficit Hyperactivity Disorder[Title/Abstract])) OR (Hyperkinetic Syndrome[Title/Abstract])) OR (Syndromes, Hyperkinetic[Title/Abstract])) OR (Attention Deficit-Hyperactivity Disorder[Title/Abstract])) OR (Attention Deficit-Hyperactivity Disorders[Title/Abstract])) OR (Deficit-Hyperactivity Disorder, Attention[Title/Abstract])) OR (Deficit-Hyperactivity Disorders, Attention[Title/Abstract])) OR (Disorder, Attention Deficit-Hyperactivity[Title/Abstract])) OR (Disorders, Attention Deficit-Hyperactivity[Title/Abstract])) OR (ADDH[Title/Abstract])) OR (Attention Deficit Hyperactivity Disorders[Title/Abstract])) OR (Attention Deficit Disorder[Title/Abstract])) OR (Attention Deficit Disorders[Title/Abstract])) OR (Deficit Disorder, Attention[Title/Abstract])) OR (Deficit Disorders, Attention[Title/Abstract])) OR (Disorder, Attention Deficit[Title/Abstract])) OR (Disorders, Attention Deficit[Title/Abstract])) OR (Brain Dysfunction, Minimal[Title/Abstract])) OR (Dysfunction, Minimal Brain[Title/Abstract])) OR (Minimal Brain Dysfunction[Title/Abstract])))

Embase

| No. | Query |
| --- | --- |
| #51 | #26 AND #50 |
| #50 | #27 OR #28 OR #29 OR #30 OR #31 OR #32 OR #33 OR #34 OR #35 OR #36 OR #37 OR #38 OR #39 OR #40 OR #41 OR #42 OR #43 OR #44 OR #45 OR #46 OR #47 OR #48 OR #49 |
| #49 | 'minimal brain dysfunction':ab,ti |
| #48 | 'dysfunction, minimal brain':ab,ti |
| #47 | 'brain dysfunction, minimal':ab,ti |
| #46 | 'disorders, attention deficit':ab,ti |
| #45 | 'disorder, attention deficit':ab,ti |
| #44 | 'deficit disorders, attention':ab,ti |
| #43 | 'deficit disorder, attention':ab,ti |
| #42 | 'attention deficit disorders':ab,ti |
| #41 | 'attention deficit disorder':ab,ti |
| #40 | 'attention deficit hyperactivity disorders':ab,ti |
| #39 | 'addh':ab,ti |
| #38 | 'disorders, attention deficit-hyperactivity':ab,ti |
| #37 | 'disorder, attention deficit-hyperactivity':ab,ti |
| #36 | 'deficit-hyperactivity disorders, attention':ab,ti |
| #35 | 'deficit-hyperactivity disorder, attention':ab,ti |
| #34 | 'attention deficit-hyperactivity disorders':ab,ti |
| #33 | 'attention deficit-hyperactivity disorder':ab,ti |
| #32 | 'syndromes, hyperkinetic':ab,ti |
| #31 | 'hyperkinetic syndrome':ab,ti |
| #30 | 'attention deficit hyperactivity disorder':ab,ti |
| #29 | 'adhd':ab,ti |
| #28 | 'attention deficit disorders with hyperactivity':ab,ti |
| #27 | 'attention deficit hyperactivity disorder'/exp |
| #26 | #1 OR #2 OR #3 OR #4 OR #5 OR #6 OR #7 OR #8 OR #9 OR #10 OR #11 OR #12 OR #13 OR #14 OR #15 OR #16 OR #17 OR #18 OR #19 OR #20 OR #21 OR #22 OR #23 OR #24 OR #25 |
| #25 | 'repetitive transcranial electrical stimulation':ab,ti |
| #24 | 'tdcss, anodal stimulation':ab,ti |
| #23 | 'tdcs, anodal stimulation':ab,ti |
| #22 | 'stimulation tdcss, anodal':ab,ti |
| #21 | 'stimulation tdcs, anodal':ab,ti |
| #20 | 'anodal stimulation tdcss':ab,ti |
| #19 | 'anodal stimulation tdcs':ab,ti |
| #18 | 'anodal stimulation transcranial direct current stimulation':ab,ti |
| #17 | 'transcranial electrical stimulations':ab,ti |
| #16 | 'stimulations, transcranial electrical':ab,ti |
| #15 | 'stimulation, transcranial electrical':ab,ti |
| #14 | 'electrical stimulations, transcranial':ab,ti |
| #13 | 'electrical stimulation, transcranial':ab,ti |
| #12 | 'transcranial electrical stimulation':ab,ti |
| #11 | 'transcranial alternating current stimulation':ab,ti |
| #10 | 'transcranial random noise stimulation':ab,ti |
| #9 | 'tdcss, cathodal stimulation':ab,ti |
| #8 | 'tdcs, cathodal stimulation':ab,ti |
| #7 | 'stimulation tdcss, cathodal':ab,ti |
| #6 | 'stimulation tdcs, cathodal':ab,ti |
| #5 | 'cathodal stimulation tdcss':ab,ti |
| #4 | 'cathodal stimulation tdcs':ab,ti |
| #3 | 'cathodal stimulation transcranial direct current stimulation':ab,ti |
| #2 | 'tdcs':ab,ti |
| #1 | 'transcranial direct current stimulation'/exp |
|  |  |

Cochrane Library

ID Search Hits

#1 MeSH descriptor: [Transcranial Direct Current Stimulation] explode all trees 1755

#2 (tDCS):ti,ab,kw OR (Cathodal Stimulation Transcranial Direct Current Stimulation):ti,ab,kw OR (Cathodal Stimulation tDCS):ti,ab,kw OR (Cathodal Stimulation tDCSs):ti,ab,kw OR (Stimulation tDCS, Cathodal):ti,ab,kw 5213

#3 (Stimulation tDCSs, Cathodal):ti,ab,kw OR (tDCS, Cathodal Stimulation):ti,ab,kw OR (tDCSs, Cathodal Stimulation):ti,ab,kw OR (Transcranial Random Noise Stimulation):ti,ab,kw OR (Transcranial Alternating Current Stimulation):ti,ab,kw 1508

#4 (Transcranial Electrical Stimulation):ti,ab,kw OR (Electrical Stimulation, Transcranial):ti,ab,kw OR (Electrical Stimulations, Transcranial):ti,ab,kw OR (Stimulation, Transcranial Electrical):ti,ab,kw OR (Stimulations, Transcranial Electrical):ti,ab,kw 1213

#5 (Transcranial Electrical Stimulations):ti,ab,kw OR (Anodal Stimulation Transcranial Direct Current Stimulation):ti,ab,kw OR (Anodal Stimulation tDCS):ti,ab,kw OR (Anodal Stimulation tDCSs):ti,ab,kw OR (Stimulation tDCS, Anodal):ti,ab,kw 2465

#6 (Stimulation tDCSs, Anodal):ti,ab,kw OR (tDCS, Anodal Stimulation):ti,ab,kw OR (tDCSs, Anodal Stimulation):ti,ab,kw OR (Repetitive Transcranial Electrical Stimulation):ti,ab,kw 2371

#7 #1 or #2 or #3 or #4 or #5 or #6 6498

#8 MeSH descriptor: [Attention Deficit Disorder with Hyperactivity] explode all trees 3475

#9 (Attention Deficit Disorders with Hyperactivity):ti,ab,kw OR (ADHD):ti,ab,kw OR (Attention Deficit Hyperactivity Disorder):ti,ab,kw OR (Hyperkinetic Syndrome):ti,ab,kw OR (Syndromes, Hyperkinetic):ti,ab,kw 7190

#10 (Attention Deficit-Hyperactivity Disorder):ti,ab,kw OR (Attention Deficit-Hyperactivity Disorders):ti,ab,kw OR (Deficit-Hyperactivity Disorder, Attention):ti,ab,kw OR (Deficit-Hyperactivity Disorders, Attention):ti,ab,kw OR (Disorder, Attention Deficit-Hyperactivity):ti,ab,kw 5290

#11 (Disorders, Attention Deficit-Hyperactivity):ti,ab,kw OR (ADDH):ti,ab,kw OR (Attention Deficit Hyperactivity Disorders):ti,ab,kw OR (Attention Deficit Disorder):ti,ab,kw OR (Attention Deficit Disorders):ti,ab,kw 7086

#12 (Deficit Disorder, Attention):ti,ab,kw OR (Deficit Disorders, Attention):ti,ab,kw OR (Disorder, Attention Deficit):ti,ab,kw OR (Disorders, Attention Deficit):ti,ab,kw OR (Brain Dysfunction, Minimal):ti,ab,kw 7232

#13 (Dysfunction, Minimal Brain):ti,ab,kw OR (Minimal Brain Dysfunction):ti,ab,kw 183

#14 #8 or #9 or #10 or #11 or #12 or #13 7944

#15 #7 and #14 122

Web of science

| # | Search |
| --- | --- |
| 1 | TS=(Transcranial Direct Current Stimulation) OR TS=(tDCS) OR TS=(Cathodal Stimulation Transcranial Direct Current Stimulation) OR TS=(Cathodal Stimulation tDCS) OR TS=(Cathodal Stimulation tDCSs) OR TS=(Stimulation tDCS, Cathodal) OR TS=(Stimulation tDCSs, Cathodal) OR TS=(tDCS, Cathodal Stimulation) OR TS=(tDCSs, Cathodal Stimulation) OR TS=(Transcranial Random Noise Stimulation) OR TS=(Transcranial Alternating Current Stimulation) OR TS=(Transcranial Electrical Stimulation) OR TS=(Electrical Stimulation, Transcranial) OR TS=(Electrical Stimulations, Transcranial) OR TS=(Stimulation, Transcranial Electrical) OR TS=(Stimulations, Transcranial Electrical) OR TS=(Transcranial Electrical Stimulations) OR TS=(Anodal Stimulation Transcranial Direct Current Stimulation) OR TS=(Anodal Stimulation tDCS) OR TS=(Anodal Stimulation tDCSs) OR TS=(Stimulation tDCS, Anodal) OR TS=(Stimulation tDCSs, Anodal) OR TS=(tDCS, Anodal Stimulation) OR TS=(tDCSs, Anodal Stimulation) OR TS=(Repetitive Transcranial Electrical Stimulation) |
| 2 | TS=(Attention Deficit Disorder with Hyperactivity) OR TS=(Attention Deficit Disorders with Hyperactivity) OR TS=(ADHD) OR TS=(Attention Deficit Hyperactivity Disorder) OR TS=(Hyperkinetic Syndrome) OR TS=(Syndromes, Hyperkinetic) OR TS=(Attention Deficit-Hyperactivity Disorder) OR TS=(Attention Deficit-Hyperactivity Disorders) OR TS=(Deficit-Hyperactivity Disorder, Attention) OR TS=(Deficit-Hyperactivity Disorders, Attention) OR TS=(Disorder, Attention Deficit-Hyperactivity) OR TS=(Disorders, Attention Deficit-Hyperactivity) OR TS=(ADDH) OR TS=(Attention Deficit Hyperactivity Disorders) OR TS=(Attention Deficit Disorder) OR TS=(Attention Deficit Disorders) OR TS=(Deficit Disorder, Attention) OR TS=(Deficit Disorders, Attention) OR TS=(Disorder, Attention Deficit) OR TS=(Disorders, Attention Deficit) OR TS=(Brain Dysfunction, Minimal) OR TS=(Dysfunction, Minimal Brain) OR TS=(Minimal Brain Dysfunction) |
| 3 | #1 AND #2 |
